# Etiology and Clinical Features of Diplopia in South China: Analysis of 303 Cases

**DOI:** 10.3389/fneur.2021.805253

**Published:** 2022-02-16

**Authors:** Zhonghao Wang, Binbin Zhu, Licheng Fu, Jianhua Yan

**Affiliations:** The State Key Laboratory of Ophthalmology, Zhongshan Ophthalmic Center, Sun Yat-sen University, Guangzhou, China

**Keywords:** diplopia, extraocular muscles, orbital diseases, cranial nerve palsy, nasopharyngeal carcinoma

## Abstract

**Purpose:**

To provide a new classification system for diplopia and evaluate the etiology and clinical features of diplopia subtypes in south China.

**Methods:**

In this retrospective study, all patients presenting with diplopia over the period from 2012 to 2014 in south China were reviewed. Patients were categorized into 3 groups according to their extraocular muscle (EOM) dysfunction: single EOM (sEOM), multiple EOMs (mEOMs), and a comitant strabismus group. Clinical data evaluated included age, sex, medical history, etiology and duration of diplopia, ocular alignment, and ocular motility.

**Results:**

A total of 303 patients were enrolled. The most common type of EOM dysfunction was sEOM (158 cases, 52.1%), followed by mEOMs (*n* = 119, 39.3%), and finally the comitant strabismus group (*n* = 26, 8.6%). Overall, the most common cause of diplopia involved orbital diseases. Within the sEOM group, microangiopathy (*n* = 42, 26.6%) and trauma (*n* = 41, 25.9%) were the major etiologies, with the lateral rectus (LR) (*n* = 86, 54.4%) being the most frequently involved. There were 12 (4.0%) patients who were considered as nasopharyngeal carcinoma (NPC)-associated diplopia (10 caused by radiation neuropathy following radiation therapy). Thyroid associated ophthalmopathy (TAO, 56 cases, 47.1%) was the predominant etiology found in the mEOMs group. Acute acquired comitant esotropia (AACE, 14 cases, 53.9%) was the most common etiology in the comitant strabismus group.

**Conclusions:**

This new classification system for assessing diplopia as based on EOM dysfunction represents an easy-to-follow approach that can be readily adapted for the clinical use. While microangiopathy and trauma represent common etiologies of diplopia, both orbital diseases and NPC-associated diplopia also warrant special attention when assessing diplopia within patients in south China.

## Introduction

Binocular diplopia (hereafter referred to as diplopia), or double vision, is a frequently encountered and challenging condition in neurological, ophthalmic, and optometric practices. Due to the wide variety of etiologies resulting in diplopia, localization of the lesion, and initial diagnosis of this condition remains a formidable task. A detail medical history and careful examinations are critical for the identification of the primary lesion site localization and final diagnosis. Considerable attention must be directed to the common diseases involving relevant cranial nerves, neuromuscular junctions, and the extraocular muscles (EOM) themselves (1, 2). Accordingly, a detailed description of representative clinical features of this condition would greatly aid clinicians in arriving at an accurate diagnosis and avoid time consuming and unnecessary investigations within the ophthalmic clinic (3). For example, with few exceptions, patients with isolated ocular motor mononeuropathies and risk factors for microvascular disease, may be safely followed clinically without neuroimaging (4, 5). Exceptions may include patients younger than 50 years of age, other neurological findings, a progressive course of diplopia, or a known history of cancer (6). This profile is based on results of histories of cases with isolated mononeuropathies in various age groups (7).

Most previous studies involved with assessing the diplopia have focused on selected populations of patients, with these cases mainly presenting within emergency medicine departments. Some typical examples consist of isolated third, fourth, and/or sixth cranial nerve palsies (8), ocular myasthenia gravis (9), and Graves' ophthalmopathy (10), all of which would not be representative of the overall profile of diplopia presenting within ophthalmologic clinics. To the best of our knowledge, no comprehensive review exists on the etiological classification and clinical features of patients who present with diplopia in south China despite its clinical importance.

As a tertiary eye center, we provide a daily service for patients with strabismus and ocular motility disorders. Patients present to our eye center with referrals from general practitioners and optometrists, as well as self-referrals, are primarily from the region of south China. Patients seen in our clinical practice are somewhat unique in that not only do they experience a variety of diplopia etiologies but also have a previous evaluating record of being seen in other hospitals. Therefore, this study was designed to review the etiologies and clinic features of patients presenting with diplopia in a tertiary eye center in south China. The coalition of such information can then provide clinicians with a template to expedite and increase the accuracy of their clinical diagnosis and treatments.

## Patients and Methods

This retrospective study was approved by the Research Ethics Board of our center and complied with the principles of the Declaration of Helsinki. Medical records of patients presenting with diplopia over the period from October 2012 to September 2014 at the Department of Strabismus and Amblyopia in a tertiary eye center in south China were retrospectively reviewed to determine the etiology and confirm the diagnosis. Patients with monocular diplopia were excluded. Cases included within the microangiopathy group consisted of those patients over the age of 50 years with vascular risk factors, such as diabetes mellitus, hypertension, hypercholesterolemia, or coronary artery disease, with no other systemic disease or history of trauma.

Clinical data on sex, age at diagnosis, laterality of the involved eye, medical history, duration of diplopia, other systemic or central nervous system diseases, ocular alignment, and ocular motility at ophthalmologic presentation as determined by a single ophthalmologist (JH Yan) were collected. Ophthalmic examinations included best corrected visual acuity, cycloplegic refraction, intraocular pressure, slit lamp biomicroscopy, fundus examination, and exophthalmometric measurement. Ocular alignment measurements were assessed using the Hirschberg corneal reflection test in the primary position and the prism alternate cover test as performed in 6 diagnostic positions. Red filter and forced duction tests were routinely performed. Patients were transferred to the Department of Neurology and Rhinology for neurologic and nasal sinus examinations or laboratory workup if they (1) were younger than 50 years, (2) lacked clear cause of ocular manifestations, (3) presented with progressive diplopia or recurrence, (4) had other neurological signs and symptoms, (5) had a history of trauma, or (6) had suspected thyroid associated ophthalmopathy (TAO). Brain and ocular imaging were routinely performed and the results were confirmed by readings from both a radiologist and an ophthalmologist. For suspected cases of myasthenia gravis, the edrophonium and/or ice-pack test, along with serological examination and electromyography were further assessed by neurologists to confirm the diagnosis.

Patients should have been classified according to the manifestations of their inadequate eye rotation in the 6 diagnostic positions corresponding to the primary functions of the 6 EOMs. However, as inadequate eye rotation in a particular diagnostic eye position did not always represent different patterns of muscle involvement, this method was only used to describe the abnormal signs of ocular motility to facilitate the classification. Therefore, we propose a new classification system for diplopia based on allocating patients into one of three groups: (1) those with limited motility in only one diagnostic gaze (a single EOM being affected, sEOM group), (2) those with limited motility in more than one diagnostic gaze (multiple EOMs being affected, mEOMs group), and (3) those with normal motility (comitant strabismus group).

### Statistical Analysis

Data analysis was performed using the IBM SPSS Statistics Version 22.4 program (SPSS, Inc., Chicago, IL, USA). To describe the etiologies and typological compositions of diplopia cohorts, the means and SDs were calculated for continuous variables, while the frequencies and percentages were calculated for categorical variables. A *p* < 0.05 was required for results to be considered as statistically significant.

## Results

Of the 303 patients included in this study, 158 (52.1%) were included in the sEOM group, 119 (39.3%) in the mEOMs group, and 26 **(**8.6%) in the comitant strabismus group. Overall, the most common cause of diplopia was trauma (23.1%), followed by TAO (19.1%), idiopathic causes (16.5%), microangiopathy (16.2%), intracerebral lesions (9.2%), myasthenia gravis (4.6%), nasopharyngeal carcinoma (NPC)-associated (4.0%), sagging eye syndrome (SES) (0.7%), myositis (3.0%), and other causes (3.6%). In an alternative classification, orbital diseases (e.g., TAO, orbital trauma, and myositis) would be considered as predominant causes. In the sEOM group, both microangiopathy and trauma accounted for approximately a quarter of the cases reviewed. In the mEOMs group nearly half (47.1%) were a result of TAO. Details regarding the etiologies of these diplopia patients are contained in Table 1.

**Table 1:** Classification and etiologies of patients with diplopia.

**Etiology**	**No. of patients**	**TAO**	**Idiopathic**	**Microangiopathy**	**Trauma**	**Intracerebral lesions**	**Myasthenia gravis**	**NPC-associated**	**SES**	**Myositis**	Other^*^
Total, *n* (%)	303 (100)	58 (19.1)	50 (16.5)	49 (16.2)	70 (23.1)	28 (9.2)	14 (4.6)	12 (4.0)	2 (0.7)	9 (3.0)	11 (3.6)
sEOM, *n* (%)	158 (52.1)	2 (1.3)	26 (16.4)	42 (26.6)	41 (25.9)	20 (12.7)	7 (4.4)	12 (7.6)	2 (1.3)	3 (1.9)	3 (1.9)
mEOMs, *n* (%)	119 (39.3)	56 (47.1)	3 (2.5)	7 (5.9)	27 (22.7)	6 (5.0)	7 (5.9)	0 (0)	0 (0)	6 (5.0)	7 (5.9)
Comitant strabismus,	26 (8.6)	0 (0)	21 (80.8)	0 (0.0)	2 (7.7)	2 (7.7)	0 (0)	0 (0)	0 (0)	0 (0)	1 (3.8)
*n* (%)								

* *1 with herpes zoster virus, 1 with Duane retraction syndorme, 2 with chronic progressive external ophthalmoplegia, 1 with conjunctival scar, 1 with high myopia, 1 after surgery of NPC, 1 after surgery of orbital tumor, 3 after intraocular lens implantation. NPC, nasopharyngeal carcinoma; mEOMs, multiple extraocular muscles; sEOM, single extraocular muscle; SES, sagging eye syndrome; TAO, Thyroid associated ophthalmopathy*.

In the sEOM group, men were predominant in all subgroups. The most affected muscle was the lateral rectus (LR) (*n* = 86, 54.4%), with the major etiologies involving the idiopathic cause and microangiopathy. More than half of these sEOM patients with superior oblique (SO) or inferior rectus (IR) perturbations were the result of trauma and microangiopathy. Three quarters of the patients with a medial rectus (MR) impairment were due to trauma. Cases in which only a superior rectus (SR) was involved were relatively rare. No case with inferior oblique (IO) involved was observed (Table 2). Among the 12 patients with NPC-associated diplopia, 10 were caused by the radiation neuropathy following radiation therapy for NPC (6 men and 4 women), and 2 were referred to ear-nose-throat department by us and subsequently diagnosed with NPC. The mean age of this sEOM group was 47.9 ± 8.0 years (range, 33–62 years). Eleven patients presented with abducens nerve palsy, with 8 showing unilateral and 1 bilateral palsy, while the remaining patient had trochlear nerve palsy. The mean time from receiving radiation therapy to ophthalmologic visit was 70.2 ± 60.1 months (range, 6–192 months) (*n* = 10) and the mean duration of diplopia was 3.4 ± 3.3 months (range, 0.4–12 months) (*n* = 12) (Table 3).

**Table 2:** Characteristics of patients with paralytic strabismus with sEOM involved.

**Affected muscle**	**Lateral rectus**	**Superior oblique**	**Inferior rectus**	**Medial rectus**	**Superior rectus**
No. of patients	86	41	18	8	5
Sex, male/female	54/32	33/8	12/6	7/1	3/2
Age, years	42.3 ± 14.2 (1–70)	46.3 ± 16.5 (20–80)	44.7 ± 21.2 (14–85)	42.9 ± 20.4 (2–63)	47.8 ± 17.4 (18–63)
Duration, months	7.9 ± 19.8 (0.1–120)	19.0 ± 35.8 (0.1–120)	17.6 ± 49.8 (0.1–216)	2.5 ± 1.8 (0.4–6)	2.7 ± 2.1 (0.8–6)
Affected eye, R/L/Both	35/43/8	13/16/9	9/8/1	6/2	4/1/0
**Cause**, ***n*** **(%)**					
TAO	0 (0)	0 (0)	1 (5.6)	0 (0)	1 (20.0)
Idiopathic	21 (24.5)	5 (12.2)	0 (0)	0 (0)	0 (0)
Microangiopathy	24 (27.9)	11 (26.9)	6 (33.3)	1 (12.5)	0 (0)
Trauma	14 (16.3)	13 (31.7)	7 (38.9)	6 (75.0)	1 (20.0)
Intracerebral lesions	10 (11.6)	7 (17.1)	1 (5.6)	0 (0)	2 (40.0)
Myasthenia gravis	2 (2.3)	1 (2.4)	3 (16.6)	0 (0)	1 (20.0)
NPC-associated	11 (12.8)	1 (2.4)	0 (0)	0 (0)	0 (0)
Myositis	2 (2.3)	0 (0)	0 (0)	1 (12.5)	0 (0)
SES	2 (2.3)	0 (0)	0 (0)	0 (0)	0 (0)
Other	0 (0)	3 (7.3)	0 (0)	0 (0)	0 (0)

*sEOM, single extraocular muscle; L, left; NPC, nasopharyngeal carcinoma; R, right; TAO, Thyroid associated ophthalmopathy*.

**Table 3:** Characteristics of individual patients with nasopharyngeal carcinoma associated diplopia.

**No**	**Sex**	**Age (years)**	**Time from radiation therapy or NPC diagnosis (months)**	**Treatment**	**Outcomes**	**Duration of diplopia (months)**	**Affected eye**	**Type of strabismus**	**Visual acuity (R/L)**	**Deviation (**°**)**
1	M	46	6	Radiotherapy	Control	2	L	Complete ANP	1.0/0.8	ET20
2	F	33	60	Radiotherapy	Control	12	R	Partial ANP	0.6/1.2	Esophoria
3	M	61	12	Radiotherapy	Control	3	L	Partial ANP	0.4/0.4	ET20
4	F	47	60	Radiotherapy	Control	3	R	Partial ANP	0.25/0.32	ET25-30
5	F	44	84	Radiotherapy	Control	2	L	Complete ANP	0.01/0.6	ET25-30
6	M	42	36	Radiotherapy	Control	0.5	R	Partial ANP	0.8/1.2	ET5-10
7	M	45	60	Radiotherapy	Control	2	L	Partial ANP	1.2/1.2	ET10-15
8	F	52	First visit with diplopia	None	None	0.4	L	Partial ANP	1.0/1.0	ET5-10
9	M	49	First visit with diplopia	Radiotherapy	Control	12	R	Partial ANP	1.0/1.0	Esophoria
10	F	51	156	Radiotherapy	Control	6	Both	Complete ANP	0.4/0.5	ET45
11	M	43	36	Radiotherapy	Control	0.7	L	Complete ANP	1.0/1.5	ET10-15
12	M	62	192	Radiotherapy	Control	6	R	SOP	1.0/1.0	Orthophoria

*ANP, abducens nerve palsy; F, Female; L, left eye; M, Male; NPC, nasopharyngeal carcinoma; R, right eye; SOP, superior oblique palsy*.

In the mEOMs group, six etiologies were identified, TAO (*n* = 56, 47.1%), oculomotor nerve palsy (ONP) (*n* = 32, 26.9%), orbital fracture (*n* = 8, 6.7%), myasthenia gravis (*n* = 7, 5.9%), myositis (*n* = 6, 5.0%), and others (*n* = 10, 8.4%). The number of men was approximately equal to that of women with regard to TAO, while there was a male dominance for ONP and orbital fractures and a female dominance for in myasthenia gravis. Most patients (73.2%) with TAO presented bilaterally. All patients with orbital fracture, 90.6% of patients with ONP, and 83.3% of patients with myositis showed unilateral impairments. Nearly all patients (56/58) with TAO presented with a dysfunction of mEOMs. Forty (71.4%) patients had a history of hyperthyroidism with the median course of 24 months (range, 1–132), while 15 (26.8%) had normal thyroid functions and thyroid function in 1 (1.8%) case was unknown. Thirteen (23.3%) patients received radioiodine therapy, 26 (46.6%) received antithyroid therapy, and 2 (3.6%) patients had undergone thyroid surgery. At their initial ophthalmologic presentation, 45 (80.4%) patients presented with normal thyroid function, 4 (7.1%) hyperthyroidism, 6 (10.7%) hypothyroidism, and 1 (1.8%) case was of unknown thyroid function. The major clinical signs consisted of eyelid swelling and eyelid retraction (35.7% for both), followed by lid lag (10.7%), proptosis (7.1%), and lagophthalmos (3.6%). An obvious enlargement of EOMs was present and both eyes were equally affected. The most frequently involved muscle was the IR, followed by SR, MR, and LR.

A complete ONP was observed in 10 (31.2%) patients and an incomplete ONP in 22 (68.8%) patients. The most common causes were trauma (59.3%), microangiopathy (18.8%), cerebral infarction (9.4%), compression from cerebral neoplasm (3.1%), and other causes (9.4%). In patients with orbital fractures, 6 were classified as blow-out orbital fractures, with orbital floor fractures accounting for 7 patients, followed by the medial wall in 4 patients and superior and lateral walls in 2 patients. In addition, two patients presented with horizontal strabismus, and 7 patients had vertical strabismus. There were 7 patients with myasthenia gravis presenting with various clinical signs, such as ptosis, vertical strabismus, esotropia, and exotropia. In patients with orbital myositis, all rectus muscles were potentially vulnerable, without the involvement of oblique muscles. Of these patients, unilateral proptosis was found in 1 patient and single muscle involvement in 2 patients, while the remaining patients experienced perturbations in two or more rectus muscles (Table 4).

**Table 4:** Characteristics of patients with paralytic strabismus with multiple EOMs involved.

**Disease**	**TAO**	**Oculomotor nerve palsy**	**Orbital fracture**	**Myasthenia gravis**	**Myositis**	**Other causes**
No. of patients	56	32	8	7	6	10
Sex, male/female	29/27	23/9	6/2	0/7	2/4	6/4
Age, years	50.9 ± 13.5 (16–86)	45.9 ± 20.8 (5–84)	29.5 ± 12.8 (10–47)	37.7 ± 25.6 (4–76)	45.3 ± 17.6 (24–71)	42.1 ± 21.0 (15–76)
Duration, months	6 (0.5–360)	2 (0.1–144)	4 (0.4–26)	12 (0.5–72)	6 (1–24)	6 (2–180)
Affected eye (R/L/Both)	6/9/41	18/11/3	5/3/0	1/3/3	2/3/1	2/2/6
Visual acuity (R)	0.93 ± 0.25 (0.4–1.5)	0.75 ± 0.39 (0.03–1.5)	7 cases >0. 8, one >0.3	0.4–1.2	0.5–1.0	0.75 ± 0.41 (0.05–1.0)
Visual acuity (L)	0.88 ± 0.26 (0.3–1.5)	0.81 ± 0.39 (0.04–1.5)	>0.8	0.4–1.2	0.5–1.0	0.94 ± 0.26 (0.05–1.2)
History/details of primary diseases, *n*	1. History of thyroid: A. Hyperthyroidism, 40, with the median disease course was 24 months (range:1-132); B. None,15; C. One was unknown 2. Thyroid dysfunction at presentation: A.Euthyroid, 45; B.Hyperthyroidism, 4; C.Hypothyroidism, 6; Unknown, 1; D.Treatment for thyroid dysfunction: radioiodine therapy, 13; antithyroid drugs, 26; and surgery,2 3. Treatment for diplopia: radioactive iodine, 1; steroid pulse therapy, 5; oral doxycycline, 2	1. Trauma,19 (traffic accident1,4; direct injury, 1; orbital trauma,4); 2. Microangiopathy, 6 (with history of hypertension and/or diabetes); 3. Cerebral infarction, 3; 4. Cerebral neoplasm,1; 5. Other causes, 3 (after surgery of nasopharyngeal carcinoma, 1; Idiopathic, 2)	Trauma, 8 (direct injury, 4; and traffic accident, 4)	Pseudophakic eye with hypertension, 1; History of Thymectomy, 1	Invovled EOMs: 1. IR L 2. SR R 3. IR+MR L 4. SR+MR R 5. IR+LR L 6. SR+IR+LR Both	1. Heavy eye syndrome, 1; 2. Chronic progressive external ophthalmoplegia, 2; 3. Post resection of pineal gland tumor, 1; 4. Cavernous hemangioma of the third ventricle, 1; 5. Conjunctival scar, 1; 6. Inferior oblique muscle palsy with diabetes, 1; 7. Duane retraction syndorme, 1; 8. After surgery of orbital tumor, 1; 9. Idiopathic,1

*Both, both eye; EOM, extraocular muscle; L, left eye; R, right eye; TAO, Thyroid associated ophthalmopathy*.

In the comitant strabismus group, 14 (53.9%) patients had acute acquired comitant esotropia (AACE), 8 (30.8%) patients had intermittent exotropia (IXT), 2 (7.7%) had acute acquired comitant exotropia (AACX), 1 (3.8%) had periodic esotropia, and 1 (3.8%) patient had dissociated vertical deviation (DVD). Related causes were found in 4 patients with AACE, such as cerebellopontine angle cholesteatoma, cerebral infarction, and head trauma, while 1 patient with DVD had a history of cataract surgery with an intraocular lens (IOL) implantation (Table 5). No clear etiologies were identified in the other 21 cases.

**Table 5:** Characteristics of comitant strabismus patients.

**Diagnosis**	**AACE**	**AACX**	**Periodic ET**	**IXT**	**DVD**
No. of patients	14	2	1	8	1
Sex, male/female	6/8	2/0	0/1	5/3	1/0
Age, years	25.1 ± 16.6 (5–65)	62, 65	39	18.6 ± 18.6 (5–59)	14
Duration, months	16.1 ± 18 (1–60)	1, 120	12	99 ± 206 (2–600)	2
Cause, *n*	Cerebellopontine angle cholesteatoma, 1; Cerebral infarction, 1; Head trauma, 2; Idiopathic, 10	Idiopathic	Idiopathic	Idiopathic	Cataract surgery with IOL implantation 1

*AACE, acute acquired comitant esotropia; AACX, acute acquired comitant extrotropia; DVD, dissociated vertical deviation; ET, esotropia; IOL, intraocular lens; IXT, intermittent exotropia*.

In idiopathic group, 26 patients had sEOM involved, 3 had mEOMs involved, and 21 had comitant strabismus. A male preponderance was observed. Mean visual acuity of either the right eye or the left eye was about 1.0. Of 29 patients with EOM affected, 26 showed unilateral impairments. Of the 21 patients with comitant strabismus, 19 had freely alternating fixation (Table 6).

**Table 6:** Characteristics of patients with idiopathic cause.

**Group**	**sEOM**	**mEOMs**	**Comitant strabismus**
No. of patients	26	3	21
Sex, male/female	21/5	3/0	13/8
Age, years	39.4 ± 12.6 (10–59)	54, 58, 74	27.2 ± 20.1 (5–65)
Duration, months	13.9 ± 48.9 (0.1–252)	0.1, 1, 5	52.5 ± 130.4 (0.3–600)
Affected eye (R/L/Both)	9/14/3	1/2/0	1/1/19 (alternating)
Visual acuity (R)	1.10 ± 0.28 (0.6–1.5)	1.0, 1.0, 1.0	0.98 ± 0.40 (0.05–1.50)
Visual acuity (L)	1.12 ± 0.30 (0.6–1.5)	0.9, 1.2, 1.0	0.98 ± 0.23 (0.60–1.50)

## Discussion

Results from the present study reveal that the most affected muscle in sEOM was the LR, followed by the SO, and IR, while the MR and SR were the least likely affected muscles. The most common causes associated with sEOM were microangiopathy and trauma similar to that of Margolin et al. (11) in which microangiopathy, trauma, and demyelinating diseases were the common causes of diplopia. Therefore, microangiopathy in the elderly and trauma in younger/middle aged cases need to be considered in patients with corresponding ocular signs. Although the exact etiologies in more than 25% of our cases with dysfunctional LR could not be determined and have been referred to as idiopathic in this report, a final diagnosis should be made only after eliminating all other possible disorders.

To date, few studies have reported NPC as a significant cause of diplopia (12, 13). NPC is an intrusive tumor with a distinct geographical global distribution (14). The world age-standardized global rate is <3 per 100,000, however, more than 70% of all new cases are documented in East and Southeast Asia. The incidence of NPC in south China is 20–30 per 100,000, much greater than that of the world average (15). In light of the high incidence of cases presenting with diplopia in our study (3.96%), strabismologists should be alerted to the possibility of NPC in patients with diplopia, in particular those from south China. Most of these NPC cases in our series (11/12) presented with complete or partial abducens nerve palsy, and only 1 case experiencing trochlear nerve palsy, which is consistent with the findings of a previous study (16). In this study, 10 of 12 cases had previously received radiation therapy. For them, the cause of diplopia was presumed to be radiation neuropathy after excluding the possibility of tumor recurrence. There are previous reports of radiation-induced cranial neuropathy cases in patients with NPC, with one study reporting a cumulative incidence of approximately 44.5% at 20 years (17). With NPC, there can be a direct compression or encroachment upon adjacent cranial nerves, with abducens nerve palsy being more readily affected due to its small size and location near the skull base where the tumor may exert its pressure (13). Therefore, these patients would be routinely referred to the Department of Rhinology for further evaluations.

It is often presumed that the involvement of mEOMs is the result of lesions (e.g., infections, tumors, and trauma) located in the areas where multiple cranial nerves may course, such as the cavernous sinus and orbital apex (18). Due to this prejudice, other bases, notably orbital disorders, such as myasthenia gravis, TAO (19), and orbital myositis (20) are often ignored. However, our current findings revealed that topping the list of causes within the mEOMs group were orbital disorders (TAO, orbital fracture, and orbital myositis). With symptoms of proptosis, eyelid retraction, mild pain within the orbit, and a history of orbital injury that are not remarkable, we have found that these patients often receive a comprehensive and unnecessary neuroimaging and systemic assessment. Under such conditions, the findings of enlarged EOMs and orbital wall fracture as detected with orbital CT/MRI are extremely important. TAO is often characterized by swollen eyelids, upper eyelid retraction, proptosis and restrictive strabismus, with the IR and MR being most commonly involved (21). Myositis represents a clinically heterogeneous, non-specific, inflammatory, and non-infectious disease (22), with the LR and SR being reported as the most frequently involved muscles (23). However, the overall results of our present study indicated that the most frequently involved muscles in myositis were the LR (*n* = 4) and IR (*n* = 4), followed by the SR (*n* = 3) and MR (*n* = 3). In addition, we found that ONP was the second leading cause in patients with mEOMs and a male preponderance was observed. The main cause of this condition was trauma, followed by microangiopathy and intracerebral lesions. As it is sometimes difficult to differentiate microangiopathy from compression by tumors based on clinical features, neuroimaging is often required for cases without any other obvious known causes (24). There was a small percentage of patients with myasthenia gravis within this mEOMs group, with all of these patients (*n* = 7) being women, most of them were young, and 1 had a history of thymectomy. Despite this low frequency (7/303, 2.3%), we still recommend that myasthenia gravis should always be given due consideration, especially in patients with ophthalmoplegia, intermittent diplopia, and ptosis.

Furthermore, an interesting finding in this study was that the frequency rate of comitant strabismus, which was observed in 8.6% of our patients. It is well-known that patients with comitant strabismus usually do not present with diplopia. However, the actual clinical evidence indicates that patients with either acute comitant strabismus or intermittent deviations, such as refractive accommodative esotropia, IXT, and DVD, might occasionally experience double vision. We found that half (*n* = 14, 53.8%) of our patients in the comitant strabismus group were AACE, with the others showing IXT, periodic esotropia, and DVD. Due to the small percentage of cases with AACE, neuroimaging is usually unnecessary for these patients.

Sagging eye syndrome is a recently recognized syndrome that involves age-related ocular connective tissue degeneration, and now considered as a cause of strabismus in the elderly (25). Extensive high-resolution MRI studies of SES have showed progressive elongation or rupture of the LR-SR band ligament, allowing inferior displacement of LR pulley. In addition, the IR pulley was displaced laterally (26). The patients with SES present progressive esotropia, cyclovertical strabismus, and limited supraduction. The external manifestations include baggy eyelids and aponeurotic blepharoptosis. Some authors proposed that neurologic evaluation and imaging might be unnecessary for SES because of its obvious external changes (27). In the current study, 2 elderly patients were diagnosed as SES who were over 60 years newly presenting with diplopia caused by esotropia (Tables 1, 2). The possibility of SES should be considered for the elderly patients with diplopia.

In previous studies, patients with diplopia were categorized into either group as based on different the cranial nerve palsies or groups based on their underlying diseases, such as TAO, myasthenia gravis, internuclear ophthalmoplegia, orbital fractures, SES, and AACE (28–30). With this categorization, there was no indication as to the direction of diplopia in these analyses (31). The results of our study provide a more robust clinical evaluation framework for use in classifying diplopia as based on EOM dysfunction. This new classification system offers an easy-to-follow approach for clinicians to assess the underlying etiologies of the diplopia and application of appropriate management strategies.

The main limitation of our study was the retrospective nature of this review and limited statistical quality due to the potential bias from some factors that might have affected our results. These factors are mainly attributable to the nature of our hospital, which is an eye center receiving a large number of referral cases of orbital diseases throughout China. Another factor was the corresponding author who was well-trained in the area of complicated strabismus with abnormal orbital structures, which may lead to case sample bias. In addition, some important clinical information regarding other systemic symptoms (e.g., dizziness, pain, fatigue, and nausea) and signs (e.g., limb sensory and motor deficits) were incomplete, which prevented us from analyzing these factors. Although this sample size was reasonable, the fact that the medical records of our patients were recorded by the corresponding author (JY), may introduce a selection bias and there was no assessment on the follow-up prognosis of these patients. Finally, as most patients in our study were Han Chinese from south China, the data obtained could introduce some ethnic and geographical bias.

In summary, to the best of our knowledge, the findings presented here represent a review containing the largest cohort of the different etiologies and relative prevalence of various subgroups of diplopia. We provided a simple framework for a new classification system in the assessment and subsequent targeted management strategies for diplopia. In light of the current data on the etiologies of diplopia as reported here, we suggest that both orbital diseases and NPC-associated diplopia should be carefully considered. Moreover, these result underline a critical role of orbital imaging in the diagnosis to avoid unnecessary systemic evaluations and possible misdiagnosis involved with other conditions.

## Data Availability Statement

The data presented in the study are deposited in the Sun Yat-sen University RDD platform, accession number 220110003, https://rdd.sysu.edu.cn/UserHome/ProjectView.aspx?AID=3971

## Ethics Statement

The studies involving human participants were reviewed and approved by the Research Ethics Board of the Zhongshan Ophthalmic Center, of Sun Yat-sen University, China. Written informed consent to participate in this study was provided by the participants' legal guardian/next of kin.

## Author Contributions

JY initially designed the concept of this work and collected the data. ZW analyzed the data. BZ wrote the manuscript. LF checked the mistakes. All authors have read and approved the manuscript.

## Conflict of Interest

The authors declare that the research was conducted in the absence of any commercial or financial relationships that could be construed as a potential conflict of interest.

## Publisher's Note

All claims expressed in this article are solely those of the authors and do not necessarily represent those of their affiliated organizations, or those of the publisher, the editors and the reviewers. Any product that may be evaluated in this article, or claim that may be made by its manufacturer, is not guaranteed or endorsed by the publisher.
